# Treatment of Atopic Dermatitis in Children

**DOI:** 10.7759/cureus.69416

**Published:** 2024-09-14

**Authors:** Faten Sid Idris

**Affiliations:** 1 Pediatrics, California Institute of Behavioral Neurosciences and Psychology, California, USA

**Keywords:** atopic dermatitis in children, chronic skin condition, eczema management, eczema treatment, inflammatory skin condition in children, moisturizing, steroids

## Abstract

Atopic dermatitis (AD), also called eczema, is a common inflammatory skin condition that causes itchy lesions and is widespread globally, especially in wealthy countries. It is the most prevalent skin disorder among children. A small percentage of children's eczema persists into adulthood, and a few of them exhibit a severe form of the condition. The development of AD is influenced by immune, environmental, and genetic factors; mutations in the filaggrin gene and a family history of atopy are regarded as risk factors that cause a hyperimmune response, which in turn increases the production of interleukin-13 and interleukin-4. As a result, the skin barrier is compromised, the T-helper 2 immune response is subsequently triggered, and atopic dermatitis develops. Diagnosing and treating AD are mostly dependent on primary care physicians. Nonetheless, treating AD is still challenging, and most pediatricians send even patients with mild eczema to dermatologists for management.

To conduct this review, we posed the central question, 'How to treat atopic dermatitis in children?' utilizing 'atopic dermatitis in children: what is the best treatment?' as the primary keywords. A comprehensive search strategy was employed, incorporating reputable databases such as PubMed, Google Scholar, and Elsevier. The inclusion criteria were set to encompass articles published in the 21st century; however, some cross-references were from the 20th century, focusing exclusively on pediatric populations worldwide. We exclusively considered articles within the realm of pediatrics to ensure relevance to our target audience, pediatricians.

The overarching objective of this review article is to elucidate the challenges encountered by pediatricians in managing even mild cases of AD and to delineate the impact on the daily lives of children and caregivers. Furthermore, this article seeks to explore the spectrum of available treatments, providing valuable insights for primary care providers to enhance the efficacy of AD management in pediatric populations.

## Introduction and background

Atopic dermatitis (AD) is a skin condition that is marked by remissions and relapses, and it is a chronic inflammatory skin condition [1]. It affects 13% [2,3] of children, with 60% of children experiencing symptoms before the age of one year and 90% by the age of five [4]. This skin disorder can compromise the integrity of the epidermal barrier due to a mix of genetic, immunologic, and environmental causes [5]. Family history of atopy and mutations in the FLG gene are two important risk factors that have been repeatedly linked to the development of AD [6]. The synthesis of profilaggrin, which is thereafter converted into filaggrin monomers, depends on the FLG gene. A deficiency in these monomers weakens the epidermal barrier, making it more susceptible to bacterial, allergenic, and irritating stimuli that cause an overreaction in the immune system [4]. Increased T-helper 2 immune response is further encouraged by increased production of interleukin-4 and interleukin-13, which impairs the epidermal barrier in AD [7].

The cause of AD has led to a debate between two main ideas: the 'outside-in' and 'inside-out' perspectives [8]. The 'outside-in' theory suggests that a weakened skin barrier, caused by problems in the skin cell development process, allows allergens to enter and trigger immune responses. Conversely, the 'inside-out' theory postulates that a specific immune system response in the skin kicks off a chain reaction leading to the development of AD [9]. Atopic dermatitis is seen as a two-phase disease driven by T cells, starting with an acute phase dominated by one type of immune signaling (Th2) and then transitioning to another (Th1) that sustains the chronic state of the condition [10].

There is no conclusive laboratory test for AD; it is a clinical diagnosis. About 80% of AD patients receive their diagnosis and treatment in a primary care environment. Diagnosing AD hinges on the clinical presentation and medical history, particularly in infants and young children [11]. Skin lesions typically manifest between two and six months, evolving from papules and papulovesicles to larger, oozing and crusting plaques. The face, hands, and extensors are the areas most commonly affected, but the scalp, neck, and trunk are also frequently involved [11]. One noteworthy observation is that, even though diaper dermatitis is common in afflicted children, AD tends to avoid the diaper area [12]. Flexural illness typically manifests itself after a year of age, affecting the wrists, ankles, and antecubital and popliteal fossae [11]. Diagnostic criteria, as outlined by the American Academy of Dermatology, include essential features such as pruritus and early age at onset. Associated features providing diagnostic support encompass atopy, xerosis, lichenification, atypical vascular responses, and various cutaneous manifestations like ichthyosis, keratosis pilaris, pityriasis alba, hyperlinear palms, ocular/periorbital alterations, and perifollicular accentuation/prurigo lesions. Recognition of these features aids in differentiating AD, fostering a comprehensive understanding crucial for effective clinical diagnosis and management [1,5].

Pruritus, or itching, is a significant symptom of eczema and is triggered by skin issues, immune system issues, and nerves [13-16]. It creates an 'itch that rash' cycle, leading to further damage and inflammation. Key factors include skin barrier problems, an abnormal immune response, increased nerve fibers, and increased sensitivity to itch. Substances like cytokines and neuropeptides influence sensory nerves, with histamine being a well-known factor. Atopic dermatitis is marked by an excess of nerve fibers, partly due to higher levels of nerve growth factor and central neural pathways involved in itch sensitization [17,18].

When diagnosing AD in children, it's crucial to consider and rule out several other conditions with similar presentations. These involve psoriasis, scabies, contact dermatitis, and seborrheic dermatitis [19]. Furthermore, AD may be mimicked by uncommon immunodeficiency-associated skin disorders such as hyper-IgE syndrome (HIES), Netherton syndrome, and Omenn syndrome. Additional clinical signs and the timing of the rash's emergence are frequently distinguishing characteristics. For example, HIES in infants might manifest as eosinophilic folliculitis, a condition that affects the face and scalp [20]. Infants with Netherton and Omenn syndromes may present with a distinctive rash in addition to recurrent diarrhea or failure to thrive, setting them apart from typical AD cases [19]. Awareness of these distinguishing factors is vital for accurate diagnosis and appropriate management of pediatric skin conditions.

Around 80% of people with AD are diagnosed and treated in a primary care practice. Primary care providers play a crucial role in recognizing and managing this common skin condition [1]. Managing AD poses challenges in pediatric primary care, with various clinical presentations complicating diagnosis and treatment. Over half of U.S. pediatricians often refer even mild AD cases to dermatologists, possibly due to discomfort in diagnosis and adherence to AD guidelines. This highlights the difficulty pediatric primary care providers may face in effectively caring for AD, even in its less severe manifestations [21-23].

In evaluating eczema, patient feedback is crucial, according to the Harmonizing Outcome Measures for Eczema (HOME) program. To gauge symptoms and determine severity, the program advises utilizing the patient-oriented eczema measure (POEM) index [24] and the most recent patient-oriented scoring atopic dermatitis (SCORAD) [25]. These tools help pediatricians track the disease's impact on young patients and adjust treatment plans accordingly, enhancing patient care [26]. Table 1 outlines the various diagnostic indexes to scale the severity of atopic dermatitis.

**Table 1:** Atopic dermatitis severity scales AD: Atopic dermatitis, BSA: Body surface area, EASI: Eczema area and severity index, IGA: Investigator’s global assessment, V-IGA: Validated investigator’s global assessment, ISGA: Investigator’s static global assessment, POEM: Patient-oriented eczema measure, PO-SCORAD: Patient-oriented scoring atopic dermatitis, SCORAD: Scoring atopic dermatitis

Diagnostic index	Description
EASI [26]	It's a tool that has been proven to be reliable and useful. The assessment looks at four specific parts of the body. It uses a 7-point scale to measure how much of the skin is affected. It considers the severity of four clinical signs such as redness or itching, on a 4-point scale. The highest possible total score on this scale is 72.
SCORAD [26,27]	This scale has been proven to be reliable in clinical settings. It uses the rule of 9s to assess how much of the body is affected by the condition. Six clinical signs (like redness or swelling) are rated on a 4-point scale to measure their intensity. It also considers what the patient reports about itching and how much sleep they've lost.
PO-SCORAD [26,28]	A scale designed for patients to assess their own condition; was developed based on SCORAD criteria, a standard for measuring skin conditions. Tailored to be user-friendly for patients and their families. Includes images and additional aids to assist in the assessment.
BSA [26]	It measures how much of the body is affected by the disease. The evaluation is expressed as a percentage of the entire body surface area (BSA).
IGA [26, 29]	A scale used to rate how severe a disease is globally. Utilizes either a 4-point or 5-point scale for assessment. Assessment is based on how the skin lesions look. The V-IGA is an example of a validated global assessment score.
POEM [24,26,30]	A score validated for reliability and accuracy. It focuses on evaluating seven specific symptoms. It evaluates symptoms throughout the last seven days. It assesses each symptom's intensity using a 5-point rating system. The highest possible total score is 35.

The burden of pediatric AD extends far beyond the physical symptoms, particularly pruritus, impacting various aspects of patients' lives and the well-being of their families. Beyond the immediate discomfort, children with AD face a higher risk of skin infections due to compromised skin barriers and immune suppression [31]. The psychosocial toll is substantial, affecting sleep quality and academic performance. Sleep disturbances, prevalent even in clinical remission, contribute to daytime drowsiness and diminished school performance [32,33]. The severity of AD correlates with the magnitude of these effects, emphasizing the pervasive nature of the condition. Socially, children may experience bullying and stigmatization, affecting self-esteem [34, 35]. Adolescents with AD may struggle with depression and psychological distress, potentially leading to a higher risk of suicidal ideation [36,37]. The burden extends to caregivers, impacting their mental and physical health [38,39]. Recognition of these wide-ranging effects underscores the importance of addressing the psychosocial dimensions of pediatric AD for both patients and their families, extending support beyond the disease's physical manifestations [26]. Recognizing the challenges faced by pediatricians in managing even mild cases of AD and the impact on daily life for children and caregivers, this article aims to explore available treatments, aiding primary care providers in effective AD management.

## Review

Therapeutic patient education

El Hachem et al. reported that according to the WHO, therapeutic patient education (TPE) is essential for managing AD and itching [40]. A multidisciplinary care team, including pediatricians, dermatologists, nurses, psychologists, and allergists, should provide tailored education and a unified approach. Comprehensive education is required on the variable clinical features of AD in a day and on one patient, its early onset, the lack of specific treatment, the sleep disturbances, the chronic nature, and potential esthetic damage. The TPE can be done with the patient alone or in a group. The procedure entails outlining the course of the illness and stressing its favorable prognosis. Personalized instructional materials, hands-on practice of dressing and massaging techniques, and coping mechanisms for itchiness and insomnia are all part of it. Psychodiagnostic testing offers treatments when necessary, and ongoing evaluation measures the patient's and family's perspective on the illness. A well-designed TPE program can lower expenditures, improve the quality of life for those with illnesses such as AD, increase control over itching and sleep disturbance, and improve medication adherence and quality [40-42]. 

Moisturizers or emollients

Frazier and Bhardwaj emphasize the importance of using fragrance-free emollients for AD treatment and prevention on a regular basis, regardless of severity [1]. This helps maintain skin moisture, reduce disease severity, and extend flare-up time [1,5,43]. According to research, emollients are recommended as the first line of prophylaxis for infants at risk of AD, particularly if they have first-degree relatives who have atopy [44,45]. For efficient skin moisture retention, emollients with high levels of lipids and a small amount of water, such as ointments, are advised. The FDA-approved medications target skin barrier dysfunction, but individual preference is crucial; no superiority has been demonstrated among the numerous over-the-counter and prescription options [46,47]. Applying emollients once or twice daily is generally recommended. Bathing recommendations include short 10-minute [5,48] regular baths, applying emollients within 3 minutes of bathing, and using diluted bleach twice weekly for antiseptic and anti-staphylococcal benefits; and wet wrap therapy, when combined with emollients and topical corticosteroids, may reduce flare-up severity [49,50].

Chow et al. emphasize the importance of using moisturizers liberally to treat AD, particularly during flare-ups, and to avoid relapses [51-54]. Doing so at least twice or three times a day is advised. They list several types of moisturizers that slow down trans-epidermal water loss (TEWL), occlusive moisturizers that create hydrophobic films, and humectants that drag and bind water to the subcutaneous layer from the deeper layers of the epidermis. Emollients fill in the spaces left by desquamating corneocytes to smooth the skin [55,56]. 'Protein rejuvenators' are small molecular weight proteins; 'therapeutic moisturizers' are moisturizers that predominately include ceramide [57]. It is recommended that moisturizer formulas be used according to the patient's climate, humidity, and environment, irrespective of the severity of their AD. Reports by van Zuuren et al. and Vieira et al. mention using traditional Asian emollients, such as virgin coconut oil [46,58]. In mild to moderate AD, camellia oil has reduced itching. Although olive oil reduced skin infection by *Staphylococcus*, it produced skin erythema. It is advised to use moisturizers after taking a bath, and in moderate to severe cases, wet-wrap therapy can improve results [59]. Eichenfield et al. do not recommend a certain frequency of baths for individuals with eczema; yet, it's crucial to eliminate any crusty skin and stay away from medicinal and alkaline soaps since they alter the natural pH of the skin [5]. Use of hypoallergenic, low-pH, and fragrance-free non-soap cleansers is recommended.

Amakye et al. report a comprehensive analysis of 72 formularies and guidelines, and recommendations were identified for 126 various kinds of leave-on emollients, with an average of 23 possibilities per formulary. All respondents gave high marks to creams and ointments; 94% said they would recommend gels, 83% lotions, and 79% other formulations. Creams regularly received the highest rating of 100% from those who used ranking systems, ahead of ointments (94%), gels (69%), lotions (41%), and others (14%). Emulsifying ointment BP (British Pharmacopoeia), Dermol 500 lotion, Dermol cream, Epimax cream, and white soft/liquid paraffin 50/50 were the top five emollients that were recommended. Notably, antimicrobial-containing Dermol 500 lotion and cream were frequently advised for short-term treatment in cases of infected eczema. Due to its sodium lauryl sulfate concentration, which is known to have negative effects on the skin, aqueous cream was frequently prohibited (six of 72 formularies). There were just 16 formularies that included aqueous cream, and the recommendations ranged from first-line use to patient demographics or self-care programs [60].

Topical steroid

Chow et al., and Frazier and Bhardwaj report that there are seven potency levels of topical steroids and emphasize the importance of selection based on the severity of AD [1-52,54,61,62]. Both sources point to the need for short-term use due to potential side effects. They also recommend twice-daily application until significant improvement [5-39,1,63,64] and a proactive approach to reduce flare-ups. Research supports the benefits of proactive treatment over-reactive treatment, particularly from Chow et al., who suggest using topical steroids once- or twice-weekly in specific affected areas [51]. Both sources emphasize the need for patient and caregiver education about steroid phobia. Frazier and Bhardwaj provide recommendations for using low-potency steroids on specific areas like the face, neck, axillae, groin, and flexor surfaces due to potential adverse effects [1]. The same article reports that there is no universally agreed-upon standard for determining the ideal quantity of topical steroids to use per application [1]. It is recommended to apply topical steroids equivalent to the size of an adult fingertip spread over an area equal to two adult palms [5]; Chow et al. introduce the concept of 'fingertip units' (FTU) to approximate the adequate dosage, considering the quantity of cream that is extruded to completely cover the distal phalanx of the index finger (Table 2). In essence, the effectiveness of topical treatment for eczema is closely tied to choosing the right formulation based on the characteristics of the affected areas [51].

**Table 2:** The FTU per age and body part The FTU concept as described by Chow et al. [5]. FTU: Fingertip unit

Area of application	Infant: 3 to 6 months	Infant: 1 to 2 years	Child: 3 to 5 years	Child: 6 to 10 years	Adolescent: 12 and above
Face/neck	1 FTU	1.5 FTU	1.5 FTU	2.5 FTU	12 FTU
Arm	1 FTU	1.5 FTU	3 FTU	2.5 FTU	3 FTU
Back	1.5 FTU	3 FTU	3.5 FTU	5 FTU	
Leg	1.5 FTU	2 FTU	4.5 FTU	4.5 FTU	6 FTU
Foot					2 FTU
Hand (both sides )					1FTU
Trunk including buttocks (front and back)					7 FTU

The recommendation is that lotion and gel are appropriate for eczema flare-ups in a hairy area with blistering and exudates. Ointments are highly recommended for thick, dry skin, like on the palms and soles. Cream is appropriate to use on all skin regions [46,51]. Table 3 shows the seven potency levels of topical steroids.

**Table 3:** The seven potency levels of topical steroids

Steroid class	Topical steroids
Class 1	Clobetasol propionate, diflorasone diacetate
Class 2	Triamcinolone acetonide, betamethasone dipropionate, amcinonide, mometasone furoate, fluocinonide
Class 3 and 4	Fluticasone propionate, mometasone furoate, triamcinolone acetonide, betamethasone valerate, fluocinolone acetonide
Class 5	Hydrocortisone valerate, hydrocortisone probutate, hydrocortisone butyrate
Class 6	Fluocinolone acetonide, alclometasone dipropionate, desonide
Class 7	Hydrocortisone, hydrocortisone acetate, dexamethasone

Topical calcineurin

Chow et al., and Frazier and Bhardwaj point out that eczema in patients aged two years and older can be treated with topical calcineurin (TCI), a steroid-sparing immunomodulator [1,51]. Tacrolimus (Protopic) and pimecrolimus (Elidel) are the main topical calcineurin inhibitors. Both articles reported that tacrolimus and pimecrolimus have emerged as effective alternatives to topical steroids in treating AD. They exhibit comparable efficacy to steroids while reducing inflammation and pruritus. These medications are recommended for patients aged two years and older. Topical calcineurin is highly recommended in cases of resistance to steroids, prolonged steroid use, parent’s steroid phobia, steroid atrophy, and when treating sensitive areas like the face, anogenital region, and skin folds [5-67]. Proactive therapy, involving the application of tacrolimus twice weekly, has shown promise in preventing symptom relapse [52-70]. Adverse effects such as burning and stinging are common, and while rare cases of skin cancer have been associated with TCI use, there is no definitive evidence establishing a causal relationship [65,71]. Despite carrying an FDA-boxed warning due to potential malignancy risks, the precise connection remains elusive, prompting ongoing research and cautious use in clinical practice.

Topical PDE4 inhibitors

Chovatiya and Paller, and Kader et al. report that in May 2016, the new topical PDE4 inhibitor crisaborole 2% ointment received a license to treat mild-to-moderate AD in people two years of age and older [5-74]; the permission was expanded to include infants as young as three months in March 2020. Crisaborole (eucrisa) addresses AD by acting as a non-steroidal anti-inflammatory agent and a PDE4 inhibitor, reducing pro-inflammatory cytokine and chemokine levels by upregulating intracellular adenosine monophosphate [73,75-76]. Frazier and Bhardwaj report that, according to a comprehensive study conducted in 2019, applying crisaborole twice a day resulted in faster lesion clearance when compared to conventional topical therapies [1,77]. Unfortunately, most patients find it financially problematic because of its exorbitant cost, which is $700 for a 60-gram tube that lasts for one month [78]. Even though crisaborole has a good safety record, there is still a need for improved topical medications due to its limited efficacy and high frequency of local application site reactions [79-81].

Janus kinase (JAK) inhibitors

Chovatiya and Paller point out that since Janus kinase (JAK) mediates Th2 cytokines like IL-4, IL-13, and IL-31, JAK inhibitors have shown promise in treating AD [72,82]. Studies have shown positive effects of JAK inhibitors in AD, including improved skin barrier function, suppression of pruritus, cutaneous nerve elongation, and impaired differentiation of Th2 cells in response to IL-4 and IL-13 [83-85]. These findings suggest that JAK inhibition holds promise as a therapeutic strategy for AD by targeting key pathways involved in inflammatory and immune responses. The FDA approves four JAK inhibitors, but none are approved for AD. However, the therapeutic class is expected to expand due to recent advancements in topical and oral JAK inhibitor development. Table 4 discusses advanced topical JAK inhibitors in the treatment of AD and their clinical trial data. Table 5 discusses advanced oral JAK inhibitors in the treatment of AD and their clinical trial data.

**Table 4:** Topical JAK inhibitors in the treatment of AD JAK: Janus kinase inhibitors, AD: Atopic dermatitis

Drugs	Class/type	FDA approval	AD efficacy	Side effects
Tofacitinib/ TOFA	First-generation JAK1/3 inhibitor	Initially FDA-approved for moderate-to-severe rheumatoid arthritis (RA) in November 2012 [86].	Reasonable efficacy in treating moderate-to-severe AD. Improving AD severity, itch, and sleep in topical form [87-89].	Includes venous thromboembolic events, major cardiac adverse events, serious infections, malignancies, and death in RA patients treated with oral TOFA.
Ruxolitinib (RUX )	A first-generation small molecule JAK1/2 inhibitor	Initially approved for myelofibrosis [90]. The New Drug Application (NDA) for topical RUX was accepted for Priority Review by the FDA in early 2021.	It has shown promise for treating mild-to-moderate AD in topical form. The RUX 1.5% and 0.75% cream BID formulations met the primary endpoint of IGA 0/1, along with key secondary endpoints of EASI-75 and clinically meaningful reduction in itch [91]. The RUX treatment was associated with improvement in itch and skin pain within 12 hours of application, number of itch-free days [92], sleep [93,94], daily activity, and work productivity [95,93].	No serious adverse events (SAEs) were reported [93]. Application site reactions were infrequent and similar to the vehicle, and no adverse events consistent with the systemic activity of RUX were detected in short-term and 44-week safety analyses.
Delgocitinib	Pan-JAK inhibitor and TyK2	It is the first topical JAK inhibitor authorized for the treatment of eczema. Treating AD in adults (January 2020) and children (March 2021, along with a 0.25% ointment) [96,97]	Efficacy and safety data were reported for pediatric patients. Potential for chronic hand eczema [98,99]. Lesion improvement and daytime and nighttime itch reduction.	Adverse events were low, with eczema herpeticum being the most common. Long-term safety data for folliculitis and acne being the most common [100, 101].
Brepocitinib (BREPO	JAK1/Tyk2 inhibitor	Under investigation for both topical and oral therapy, including its potential application in AD.	Efficacy was observed in individuals aged 12 to 75 years with mild to moderate eczema [102].	Well-tolerated/primary safety outcomes met [103].

**Table 5:** Oral JAK inhibitors in the treatment of AD JAK: Janus kinase inhibitor, AD: Atopic dermatitis, URTI: Upper respiratory tract infection, CPK: Creatine phosphokinase

Drugs	Class/type	FDA approval	AD efficacy	Side effects
Baricitinib (BARI)	First-generation oral JAK1/2 selective inhibitor	FDA approved it in June 2018 for adult moderate-to-severe rheumatoid arthritis inadequately responsive to TNFα inhibitors [104]. Subsequently, it was approved for adults with moderate and severe AD.	Authorized for moderate and severe AD [105]	Nasopharyngitis, URTI, folliculitis, and herpes infection were more common. Laboratory abnormalities included increased blood CPK and elevated lipids, with minimal changes in hematologic, hepatic, and renal parameters. Malignancies were absent. Severe events were less common [106,107].
Upadacitinib (UPA)	JAK1-selective inhibitor	Initially FDA-approved in August 2019 for the management of moderate and severe rheumatoid arthritis in adults. A supplemental new drug application (sNDA) for UPA in adults (30 and 15 mg QD) and adolescents (15 mg QD) with moderate-to-severe AD was submitted to the FDA in late 2020 [72].	Showed efficacy in AD. Effective as monotherapy and in combination with topical corticosteroid therapy (TCS). Rapid response was observed, with significant improvements in skin clearance and itch as early as week 2 and week 1, respectively [72].	Common adverse events (AEs) included URTI, acne, worsening AD, headache, increased blood CPK, nasopharyngitis, and nausea [72].
Abrocitinib (ABRO)	JAK1-selective inhibitor	A new drug application (NDA) for ABRO (200 or 100 mg QD) for adults and adolescents 12 years and with moderate-to-severe AD was accepted to undergo priority review by the FDA in late 2020.	Demonstrated noteworthy effectiveness in treating people with moderate-to-severe AD for ABRO 200 mg and 100 mg [108]. Phase 3 monotherapy trials demonstrated effectiveness in subjects ≥12 years with moderate-to-severe AD.	Common adverse events (AEs) included AD, URTI, headache, nausea, and diarrhea [109].
Gusacitinib (GUSA)	Inhibitor of spleen tyrosine kinase (SYK) and JAK	The FDA authorized the medication in February 2021 for moderate-to-severe chronic hand eczema.	Early results from a randomized, double-blind, placebo-controlled trial showed that the medication was well tolerated and produced positive safety and efficacy outcomes.	The placebo group witnessed a decreased incidence of treatment-related side effects, such as headache, nausea, and diarrhea [110].

Systemic therapy

Eichenfield et al. reported that systemic therapy may be necessary for approximately one-third of young children with moderate-to-severe AD who do not respond to topical medication and phototherapy [26,4,111]. Systemic immunomodulators are advised for moderate-to-severe AD according to current guidelines [73,112,113] yet very few have been granted licenses for this use. Dupilumab, a targeted biologic treatment, has been approved for children with moderate-to-severe, poorly managed eczema ages six to 11 [114,115]. Off-label drugs such as methotrexate, cyclosporine, mycophenolate mofetil, and azathioprine are also taken into account [73,112,4]. There is insufficient data to support the use of systemic antibiotics as a preventive measure in AD settings or to show that topical or oral antibiotics improve the condition of eczema-affected children [116]. On the other hand, secondary bacterial infections have been effectively treated with oral antibiotics [4,112,114] While there is little evidence to support the use of oral antihistamines for treating AD, urticaria may be treated with nonsedating antihistamines, and patients experiencing pruritus and trouble sleeping may benefit from sedating antihistamines (Table 6) [5,1,114].

**Table 6:** Systemic therapy AD: Atopic dermatitis

Type	Description
Antibiotics	Atopic dermatitis is a condition that disrupts the epidermis and increases the risk of skin infections; 90% of patients are colonized with Staphylococcus aureus [ 49]. A study found that bleach baths and intranasal mupirocin reduced the severity of the condition in three months [49]. Yet there's no conclusive data to back up the prophylactic usage of oral antibiotics. However, oral antibiotics as a prophylactic measure, are not well-supported by research, as they are only intended for secondary bacterial infections [ 4,117,118].
Antihistaminics	The recommendation of oral antihistamines for reducing pruritus in AD is insufficient, and patients experiencing sleep disruptions may be prescribed short-term, sporadic courses of oral sedating antihistamines like hydroxyzine or diphenhydramine (Benadryl). Topical antihistamines are not advised due to potential contact dermatitis risks [5].
Dupilumab	This is a monoclonal antibody with FDA approval that targets the IL-4 and IL-13 receptors to treat people with moderate-to-severe AD [119,120]. Rapid improvements in disease severity and extension were observed in two randomized, double-blind, placebo-controlled trials. It is useful in combination with topical corticosteroids (TCSs) to improve AD symptoms [121]. Ocular adverse reactions such as blepharitis, noninfectious conjunctivitis, and dry eyes are frequently reported [122]. Dupilumab is considered safe and doesn't need to be monitored in a lab.
Systemic glucosteroids	Although systemic glucocorticoids are FDA-approved and usually effective, they are rarely advised as an interim treatment for AD. They are advised for brief courses in particular circumstances (e.g., quick relief of acute flares or switching to steroid-sparing treatment) due to a lack of high-quality randomized controlled trials. Guideline recommendations suggest starting at 0.5 mg/kg/day for one to two weeks [123,124], followed by a steady decrease over a month. There are serious hazards involved with long-term use, thus it is important to look into other therapeutic choices [114, 125].
Methotrexate (MTX)	Methotrexate is an effective chemotherapeutic drug for adults and children with moderate-to-severe eczema. It's advised as a third-line therapy since it prevents DNA, RNA, and purine production by inhibiting dihydrofolate reductase. In people with severe AD, MTX has demonstrated comparable efficacy to azathioprine and low-dose cyclosporine. To reduce toxicities, initial doses of 7.5 to 25 mg per week are advised, along with daily folic acid [4,126].
Mycophenolate mofetil	When other immunosuppressants are ineffective or unsuitable for treating moderate-to-severe AD, mycophenolate mofetil (MMF) and mycophenolate sodium may be used as third-line treatments. Fewer trials have produced encouraging findings, with a notable reduction in the severity of the illness [127].
Azathioprine (AZA)	For individuals with moderate or severe eczema, it is strongly advised as a third line of defense. It effectively suppresses purine synthesis, which has a direct impact on DNA synthesis. Azathioprine is comparable to methotrexate in its efficacy. One to three milligrams per kilogram per day is the recommended dosage [128], and vomiting and nausea are possible side effects [4,129].
Cyclosporine (CsA)	Many nations have authorized the use of the oral calcineurin inhibitor CsA to treat AD. It prevents cytokine transcription and lowers inflammation by suppressing T-cell transcription factor (NF-AT). Reliable randomized controlled trials demonstrate that CsA is superior to immunoglobulin, prednisolone, and phototherapy in improving AD by 50% to 95%. The recommended starting dose is 3-6 mg/kg/day, according to the American Academy of Dermatology [4,129,130]. Nephrotoxicity and hypertension are frequent adverse consequences [4].

Other treatments options

Phototherapy

Kader et al. report that for severe AD unresponsive to topical corticosteroids, ultraviolet B (UVB) phototherapy, including narrow-band and broad-band UVB, UVA, UVA1, and UVB, is recommended. While there's no skin cancer risk, UV exposure is necessary, demanding two to three sessions weekly for several months. Combining psoralen with phototherapy rapidly reduces pruritus within two weeks. Phototherapy has multifaceted benefits, such as suppressing Staphylococcus aureus colonization, thickening the stratum corneum, and modulating immune responses, contributing to immunosuppression [73].

Integrative Medicine

Chow et al. point out that Asian cultures have a long history of using complementary and alternative remedies for AD. However, since there isn't much data to support their frequent use, vigilance is needed. Conventional herbs, which are frequently used, can include unidentified contaminants that may interact with or induce hypersensitive reactions. Studies involving acupuncture and acupressure are limited because they don't have placebos or controls. The stress-relieving effects of massage and aromatherapy are influenced by counseling effects. Even though traditional herbs have been investigated extensively, convincing data still needs rigorous multinational trials that concentrate on side effects and quality of life. During doctor visits, patients should be made aware of the inadequate research on these treatments and be encouraged to discuss how they can be used [51].

Vitamin D

Mansour et al. state that the supplemented group exhibited a larger percentage change in the eczema area and severity index (EASI) score, a substantial increase in their 25-hydroxy vitamin D levels, and a drop in their EASI score compared with the placebo group. This suggests that vitamin D could be a beneficial additional treatment for severe AD. However, further research, especially multicenter studies with diverse populations, is needed to confirm these results and investigate the potential long-term benefits, especially during winter-related eczema [131].

Results

**Figure 1:**
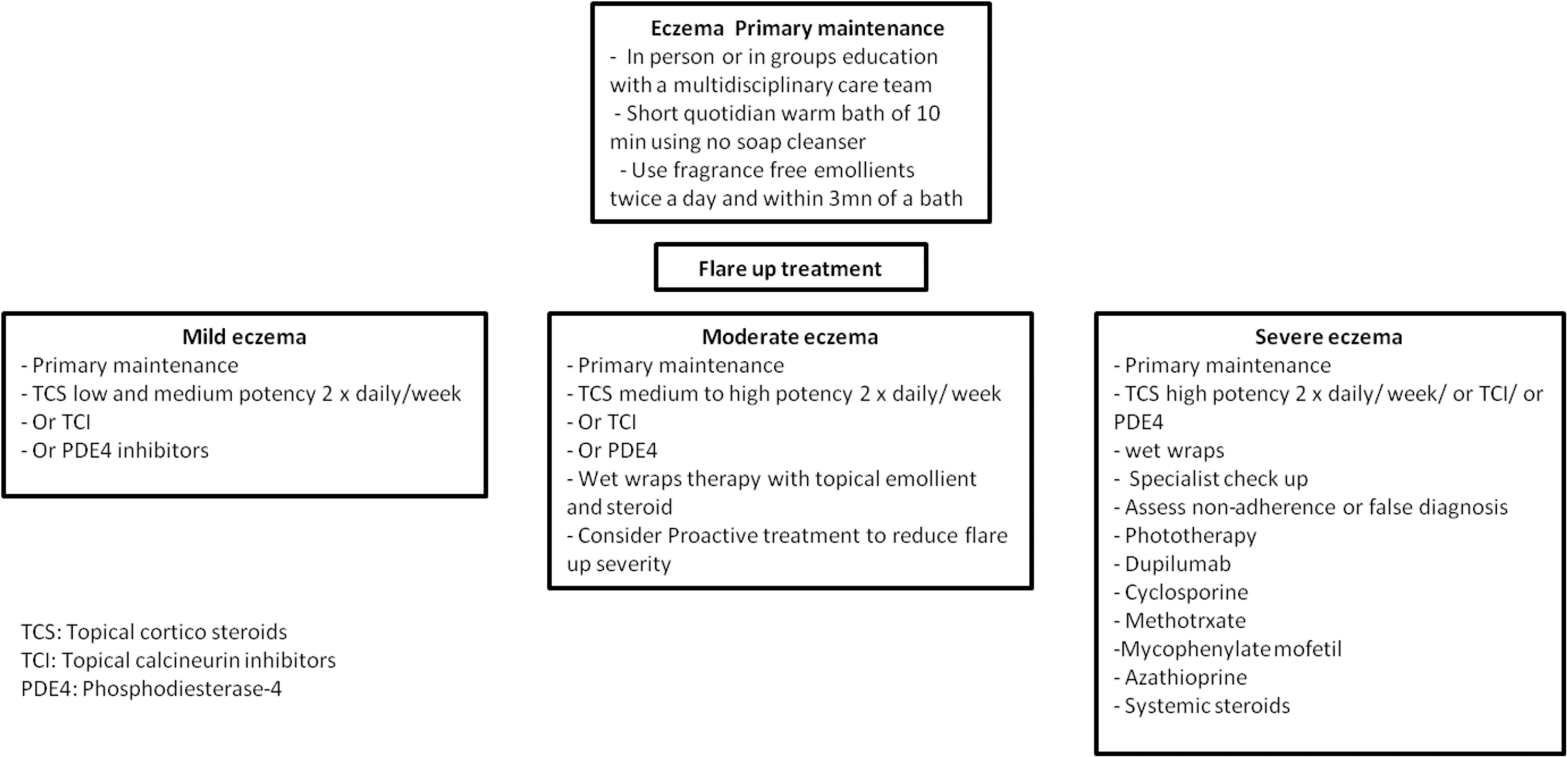
Atopic dermatitis treatment

Figure 1 summarizes the treatments for AD that were identified by the research conducted for this review article.

## Conclusions

In summary, treating AD in children requires more than just administering medication. It also calls for a thorough understanding of the physiopathology of the illness and a patient-centered approach that takes into account social and psychological issues in addition to physical symptoms. This review emphasizes the importance of clear communication and education while highlighting the basic therapies for AD that are sometimes disregarded. The myths and anxieties related to topical corticosteroids are dispelled, emphasizing their effectiveness, particularly in treating flare-ups. By closing the knowledge gap, medical professionals can enable parents and other caregivers to carefully administer these therapies, reducing the financial burden that this chronic illness places on children and their families.

The paper also highlights the need for TPE and the critical function of a multidisciplinary treatment team. Given that AD has a variety of clinical characteristics, an early onset, and is a chronic condition, specialized education is essential. It is recommended that training be given regarding this condition for primary care physicians while continuously educating parents and children to lessen the daily difficulties that AD presents. This would improve treatment compliance and foster teamwork in addressing sleep disorders, poor academic performance, bullying, and possible cosmetic harm related to AD.
